# Geospatial analysis of blindness within rural and urban counties

**DOI:** 10.1371/journal.pone.0275807

**Published:** 2022-10-10

**Authors:** Facundo G. Sanchez, Stuart K. Gardiner, Shaban Demirel, Jack P. Rees, Steven L. Mansberger

**Affiliations:** 1 Legacy Devers Eye Institute, Portland, Oregon, United States of America; 2 Discoveries in Sight Research Laboratories, Portland, Oregon, United States of America; Xiang’an Hospital of Xiamen University, CHINA

## Abstract

**Purpose:**

To determine the associations of blindness within rural and urban counties using a registry of blind persons and geospatial analytics.

**Methods:**

We used the Oregon Commission for the Blind registry to determine the number of persons who are legally blind, as well as licensure data to determine the density of eye care providers (optometrists and ophthalmologists) within each county of the State of Oregon. We used geospatial statistics, analysis of variance, and logistic regression to determine the explanatory variables associated with blindness within counties.

**Results:**

We included 8350 individuals who are legally blind within the state of Oregon in the calendar year 2015. The mean observed prevalence of registered blindness was 0.21% and ranged almost 9-fold from 0.04% to 0.58% among counties (p < .001). In univariate models, higher blindness was associated with increasing median age (p = .027), minority race (p < .001), decreased median household income (p < .001), increased poverty within a county (p < .001), and higher density of ophthalmologists (p = .003). Density of optometrists was not associated with prevalence of blindness (p = .89). The final multivariable model showed higher blindness to be associated with lower median household income, higher proportion of black race, and lower proportion of Hispanic race (p < .001 for all).

**Conclusion:**

Geospatial analytics identified counties with higher and lower than expected proportions of blindness even when adjusted for sociodemographic factors. Clinicians and researchers may use the methods and results of this study to better understand the distribution of individuals with blindness and the associated factors to help design public health interventions.

## Introduction

Blindness is a major cause of disability worldwide with a high economic cost ($139 billion, 2013) [1]. A nationwide study (2015) estimated a total of 4.24 million people with low vision or blindness with a projected increase to 8.9 million by 2050 [2]. This projected increase will create demand on public health resources that may vary widely from state to state. This variability within states may be related to access to eye care, and sociodemographics [3–9]. Decreased access to health care may be common in rural areas [10]. Oregon has a large rural and urban population (with counties ranging from 0.7 to 1626 people per square mile) with variable climates and ethnicities (e.g. ranging from 1.4 to 33.5% Hispanic and from 0.2% to 16.1% Native American). Interestingly, we found only a single publication that reports the prevalence of blindness in rural areas in the United States (US) [11].

Blindness registries are common within the US. In contrast to self-report survey data (e.g. Behavioral Risk Factor Surveillance System [12] or the American Community Survey [13]), blindness registries require than an eye care provider certify that a person meets the criteria for blindness. Once entered, the registry members are eligible for state and national benefits such as social security benefits, and reduced taxes and utility rates [14]. However, the rules for determining disability are complicated, and applying for disability benefits can be a difficult and lengthy experience for individuals [15].

We used a blindness registry to determine the proportion of blindness and the variation of blindness within rural and urban counties. We also determined the density of eye care providers (optometrists and ophthalmologists) within each county. We hypothesized that there would be a high variability in blindness registration within the state that may be explained by access to eye care [16]. To our knowledge, this is the first study to use geospatial statistics and county-level data to examine the epidemiology of blindness within a state. Researchers, eye care providers, and public health representatives may use this information and similar methods to identify counties that would benefit from public health interventions to decrease blindness.

## Methods

The Legacy Health Institutional Review Board (Portland, OR) waived the requirement for informed consent and approved this study of de-identified data. All aspects of the study adhered to the tenets of the Declaration of Helsinki. All data were fully de-identified before conducting the study.

### Prevalence of blindness

We used a registry from the Oregon Commission for the Blind (OCB; Portland, OR) to develop a database of blind individuals in the State of Oregon. The OCB is the state agency in Oregon that maintains a registry of individuals who have been diagnosed as legally blind, defined by US law as best-corrected visual acuity equal to or worse than 20/200, or a visual field of less than 20 degrees in the better seeing eye [17]. The OCB receives monthly updates from the Oregon State Department of Administrative Services (DAS), which in turn collects candidates for the registry from the Department of Motor Vehicles (DMV), Oregon Vocational Rehabilitation Services, disability insurance, Veterans Affairs (VA), and other health care agencies. Prior to entry into the OCB registry as legally blind, an OCB ophthalmologist reviews the health records of each new submission to confirm eligibility or determine the need for additional examinations. The OCB registry includes: date entered in the OCB registry, age, gender, race, age of onset and cause of blindness, city and county of residence. The OCB regularly updates the database to remove those deceased or moved out of state. We calculated the prevalence of blindness per 100,000 persons within a county by dividing the number of individuals who were blind within each of Oregon’s 36 counties by the county’s population in 2015 according to the annual Census Bureau’s Population Estimates Program and multiplying this proportion by 100,000 [18].

### Density of eye care providers

We created a database of active ophthalmologists and optometrists as of 2015 from the Oregon Medical Board and the Oregon Board of Optometry, respectively, through a public record request. The database contained the providers’ name, license status, and practice street address. We excluded providers marked as inactive in their respective databases. We calculated the density (per 100,000 persons) of eye care providers (optometrists and ophthalmologists together and separately) by dividing the number of eye care providers by the county population and multiplying this proportion by 100,000.

### Odds of blindness by county

We determined the odds of blindness (i.e. the probability that an individual is blind divided by the probability that they are not blind) within a county using a multivariable model as follows. First, we used univariate analysis with county level data of age, gender, race/ethnicity, median household income, and poverty rate according to the American Community Survey 5-Year Estimates [19]; and the urban-rural classification according to the National Center for Health Statistics (NCHS) [20]. The NCHS uses a 6-level scale for the rural-urban classification, where 1 represents a "large central metro county", and 6 a "non-core county". Candidate variables with p<0.2 in these univariate analyses were then included in an initial multivariable model. Lastly, we used single backward elimination procedure (p>0.10) to determine a final multivariable model. The final model predicted the odds of blindness within each county based on socio-demographic factors. Next, we determined whether the density of providers was related to the observed prevalence of blindness after adjusting for the socio-demographic factors. We performed this analysis for odds of blindness overall, and separately for several disease-specific causes of blindness, using the same multivariable model in each case.

### Geospatial data analysis

We used logistic regression models to predict each county’s prevalence of blindness, weighting observations according to the population of each county (since prevalence estimates when expressed as a proportion can be considered to be more accurate in counties with higher population). We further assumed an exponential spatial correlation structure, such that the correlation between residuals from the model decreased exponentially with the Euclidean distance between the geographical centers of the counties, to account for the likelihood that some residents would cross to a nearby county to receive care. We performed all analyses in R (version 3.5.0, R Foundation for Statistical Computing, Vienna, Austria), using the glmmPQL function from the MASS core package and a Gaussian random effect with constant grouping parameter. We excluded conditions if they were responsible for less than 10 individuals with blindness statewide, and/or that did not have more than one blind individual within any single county, since there was not sufficient statistical power to detect any county-level differences for these conditions. When location of the blind individual was not available, we imputed this information using the ‘MICE’ package (version 3.8.0). The MICE package creates imputations (replacement values) for multivariate missing data [21].

We used the United States Geological Survey (USGS) National Map Viewer (public domain) to create the Oregon Map in the Figure [22] with Paintbrush (Version 2.6–20210402) to produce the grayscale in the images. County data (median household income and race/ethnicity) were obtained from the American Community Survey 5-Year Estimates [18, 19]. Ophthalmologists data were obtained via Public Record Request to the Oregon Medical Board (2015) [23]. Optometrists data were obtained via Public Record Request to the Oregon Board of Optometry (2015) [24]. Blindness data were obtained from the Oregon Commission for the Blind Public Record Request [25]. Population estimates for 2015 were obtained from the U.S. Census Bureau, Annual Census Bureau’s Population Estimates Program for Oregon [18].

## Results

### Population of Oregon and the prevalence of blindness

Table 1 displays sociodemographic and economic variables, blindness, and eyecare provider density in Oregon counties. The 2015 census population estimate of Oregon demonstrates a total population of 3.9 million, where 3.3 million individuals live in 13 urban counties and only 0.65 million in 23 rural counties. Furthermore, over 60% of the Oregon population is concentrated in only 5 counties (Marion, Multnomah, Washington, Clackamas, and Lane) located in the West of the state across the Willamette Valley.

**Table 1:** Sociodemographic and economic variables, blindness, and eyecare provider density.

County	Median age	Females (%)	White race (%)	Black race (%)	Hispanic race (%)	Population	Urban designation (NCHS)*	Poverty (%)	Median Household Income	Total individuals with blindness**	Individuals with blindness***	N of ophthalmologists***	N of optometrists***
**Oregon State**	39.1	50.1	72.6	2.4	11.6	3939233	N/A	14.5	46969	**8350**	237.1	4.5	14.9
Baker	48.2	49.1	92.1	0.3	3.8	16052	0	15.3	43765	**47**	292.8	6.2	18.7
Benton	32.7	49.6	77.2	0.8	6.5	86495	1	20.7	54682	**126**	145.7	2.3	15.0
Clackamas	41.3	50.8	78.1	0.9	7.7	389438	1	9	72408	**494**	126.8	7.2	14.9
Clatsop	43.9	50.4	81.2	0.7	7.6	37382	0	12.2	49828	**85**	227.4	5.4	18.7
Columbia	42.9	50	84.4	0.4	4.3	49389	1	12.3	57449	**104**	210.6	0.0	12.1
Coos	48.1	50.9	83.6	1.1	5.8	62775	0	17.9	40848	**230**	366.4	8.0	12.7
Crook	48.1	50.6	77.9	0.5	6.5	20956	0	15.3	41777	**58**	276.8	0.0	0.0
Curry	54.6	51.3	85.7	0.3	6.2	22338	0	15.5	42519	**66**	295.5	9.0	22.4
Deschutes	41.9	50.7	76.3	0.4	6.7	166622	1	12.1	59152	**263**	157.8	8.4	27.0
Douglas	46.9	50.6	86.3	0.3	5.0	107194	0	17	44023	**355**	331.2	6.5	16.8
Gilliam	48.2	50.1	89.6	1.0	7.8	1883	0	9.9	39831	**11**	584.2	0.0	0.0
Grant	51.1	50.6	93.5	0.3	3.5	7276	0	13.7	44826	**20**	274.9	0.0	13.7
Harney	46.2	49.8	86.6	0.6	4.6	7229	0	17.5	39504	**15**	207.5	0.0	13.8
Hood River	39.4	50.2	63.0	0.5	29.7	22749	0	12.1	57269	**31**	136.3	8.8	26.4
Jackson	42.8	51.3	78.2	0.7	11.2	208363	1	16.7	48688	**615**	295.2	10.6	23.0
Jefferson	39.9	48.7	55.3	0.9	17.9	22061	0	20.9	48464	**31**	140.5	0.0	9.1
Josephine	47.9	51	83.8	0.3	6.6	83409	1	18.6	40705	**307**	368.1	9.6	8.4
Klamath	42.5	50.3	77.7	0.9	11.3	65972	0	18.7	42531	**85**	128.8	7.6	7.6
Lake	48.3	46.4	85.2	0.6	7.7	7842	0	20	32769	**16**	204.0	0.0	12.8
Lane	39.3	50.9	78.4	1.0	7.6	357060	1	18.8	47710	**1208**	338.3	6.7	14.8
Lincoln	50.4	51.3	78.1	0.3	7.9	46347	0	18.4	43291	**93**	200.7	6.5	19.4
Linn	39.5	50.6	80.7	0.4	7.7	118971	1	16.1	49515	**221**	185.8	4.2	16.8
Malheur	36.1	45.3	61.9	1.2	32.5	30551	0	25.2	37112	**68**	222.6	16.4	22.9
Marion	35.8	50.2	62.4	1.0	23.6	323259	1	15.9	53828	**552**	170.8	5.9	14.2
Morrow	37.4	48.2	61.0	0.4	33.5	11204	0	14.7	54386	**4**	35.7	0.0	0.0
Multnomah	36.5	50.6	67.5	5.2	10.5	768418	1	16.4	60369	**2064**	268.6	18.0	23.9
Polk	37.4	51.9	72.2	0.6	11.6	77264	1	15.4	56032	**110**	142.4	0.0	10.4
Sherman	49.8	49.7	89.3	0.6	7.5	1795	0	13.7	42074	**5**	278.6	0.0	0.0
Tillamook	48	49.4	81.0	0.2	9.5	25430	0	15.5	45061	**42**	165.2	0.0	19.7
Umatilla	36	47.9	67.1	0.7	25.0	76738	0	17.8	50071	**107**	139.4	3.9	14.3
Union	40	50.8	87.3	0.7	4.3	25745	0	17.4	46228	**117**	454.5	3.9	19.4
Wallowa	52.2	50.9	90.6	0.3	2.6	6857	0	13.7	44877	**31**	452.1	0.0	29.2
Wasco	41	50.4	73.1	0.6	15.9	25492	0	13.7	48510	**42**	164.8	0.0	19.6
Washington	36.1	50.8	63.5	1.7	15.0	556210	1	10.3	74033	**577**	103.7	5.6	28.0
Wheeler	56.5	51.5	94.5	0.0	1.4	1348	0	20.6	33563	**4**	296.7	0.0	0.0
Yamhill	38.2	49.9	73.9	1.2	14.6	101119	1	13.7	58392	**146**	144.4	3.0	9.9

* Urban designation according to the National Center for Health Statistics (NCHS).

** 33.3% of the individuals with blindness contain imputed geolocation data.

*** Per 100,000 persons. N/A: Not applicable.

**Fig 1:**
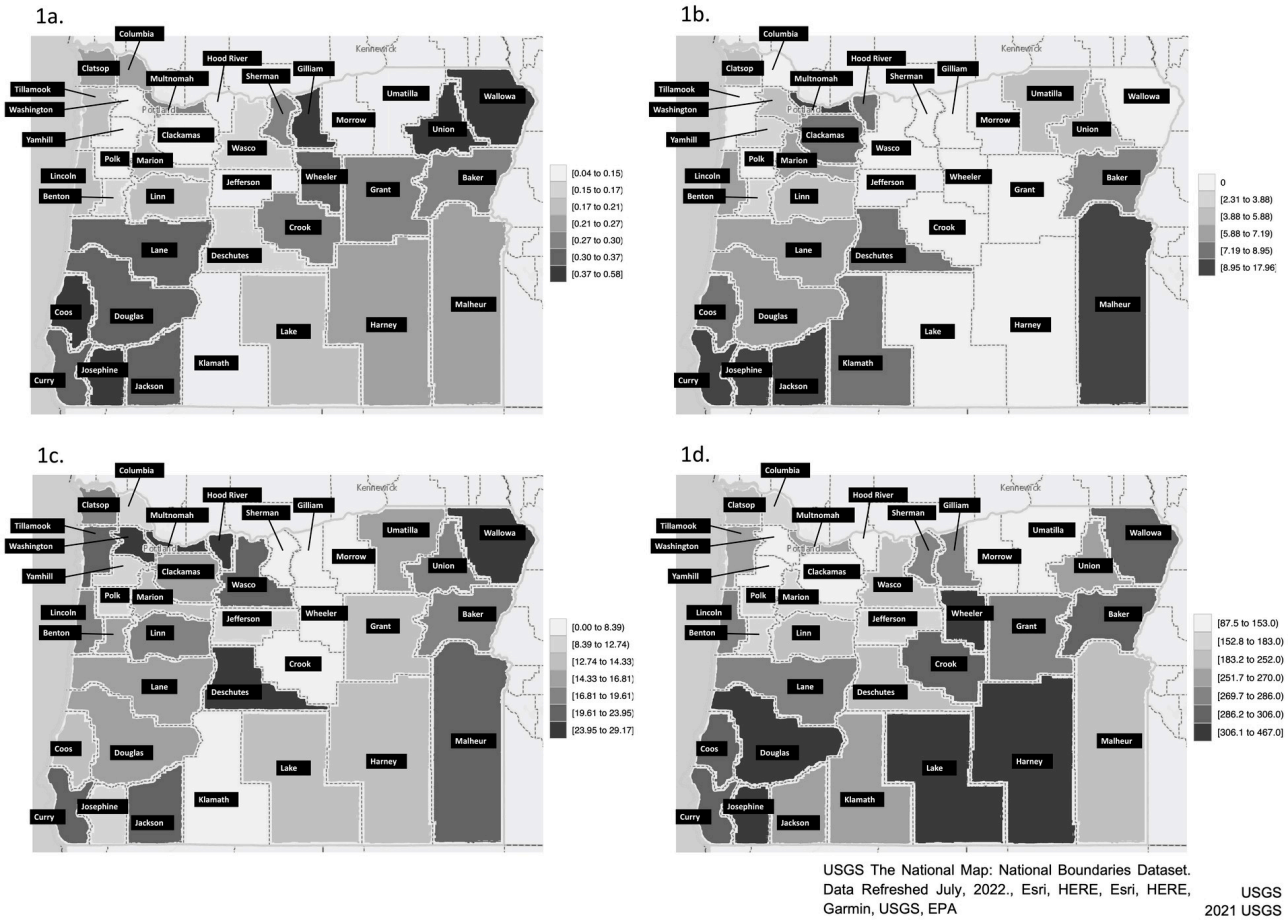
**a**. Prevalence of registered individuals with blindness per county in Oregon. **b**. Number of ophthalmologists in each Oregon county per 100,000 persons (year 2015). Counties with higher densities of ophthalmologists registered more people with blindness from any cause (OR 6.5 for blindness with one more ophthalmologist, p = .003, in a multivariable model using county data including median household income and race/ethnicity). **c**. Number of optometrists in each Oregon county per 100,000 persons (year 2015). The density of optometrists was not associated with blindness (p = .889) in a multivariable model using county data including median household income and race/ethnicity. **d**. Multivariable model for the odds of blindness per 10,000 persons by county (year 2015). Multivariable model using county data (median household income and race/ethnicity) in addition to density of ophthalmologists to predict the odds of blindness per 100,000 persons by county.

Fig 1a show the prevalence of blindness in the registry by county. Overall, registered blind individuals represented 0.21% of the state’s population in 2015. Between counties, the observed proportion of blindness in the registry ranged from 0.04% to 0.58% (p < .001 for differences between counties). The OCB registry included 8350 individuals who were blind, with geospatial information available for 5570 (66.7%). The subset that did not include geolocation data were younger on average (53.63±27.28 vs. 58.53±26.43 years, p < .001); but the other demographic and socioeconomic variables (gender, race/ethnicity, cause of blindness, and location) did not differ significantly (all p>.05). The results were similar with and without imputation, and therefore we show the results for the data including imputation.

### Density of eye care providers

Fig 1b display density of ophthalmologists in Oregon and show a wide difference in density per 100,000 persons. Overall, the average number of ophthalmologists in a county was 4.54 per 100,000 persons (SD 4.71, range 0–17.96). A large proportion of Oregon counties (38.9%, 14/36) had no ophthalmologist (Columbia, Crook, Gilliam, Grant, Harney, Jefferson, Lake, Morrow, Polk, Sherman, Tillamook, Wallowa, Wasco, and Wheeler), which represented 6.80% (283,767 individuals) of the Oregon population; and 12 of the 14 counties (86.7%) were rural. The density of ophthalmologists in rural versus urban counties was 40.5% lower (3.48 versus 5.85 per 100,000 persons, respectively). Fig 1c displays the density of optometrists and also shows large differences in density between counties. The density of optometrists was more than 3 times higher than the density of ophthalmologists at 14.88 (8.20, 0–29.17), and only five counties (13.9%, 5/36) (Crook, Gilliam, Morrow, Sherman and Wheeler) had no optometrists. All these 5 counties were rural, and the density of optometrists in rural versus urban counties was 14.3% lower (13.40 versus 15.63 versus 100,000 persons). These counties also had no ophthalmologist and the population represented 0.96% of the state population. Overall, this suggests that about 7% and 1% of Oregonians had poor access within their county to ophthalmologists and optometrists, respectively.

### Univariate and multivariate associations of blindness

Table 2 shows the univariate and multivariable analysis for demographic and socioeconomic predictors. Blindness was associated with increasing age (p = .027), race/ethnicity (p < .001), decreased median household income (p < .001), and increased poverty within a county (p < .001) in a univariate model. Black race was associated with higher prevalence of blindness (OR 1.13, p< .001), while counties with higher proportions of Hispanic individuals were less likely to have persons with blindness in the registry (OR 0.97, p = .01). Blindness was not associated with the rural-urban classification, independently of considering it a binary (rural-urban), or categorical variable (using the 6 categories) (p>.2 for both). The final multivariable model showed a higher proportion of blindness to be associated with decreased county median household income, higher proportion of black race, and lower proportion of Hispanic race (OR 0.67, 1.07, and 0.98, respectively; p < .001 for all).

**Table 2:** Socioeconomic and demographic predictors of the prevalence of blindness.

		Univariate Analysis*		Multivariable Analysis*	
		Odds Ratio	p-value	Odds Ratio	p-value
Median Age (per year older)		1.04	0.027		
Gender (%)		1.00	0.606		
Urban County (vs. Rural, NIHS)		0.83	0.334		
Median household Income (per $10,000 higher)		0.76	<0.001	0.67	<0.001
Poverty (per 1% higher)		1.10	<0.001		
Race/Ethnicity (per 1% higher proportion of population)	Black	1.13	<0.001	1.07	<0.001
	Asian	0.95	0.073		
	Native American / Alaskan	1.00	0.986		
	Pacific Islander	0.88	0.712		
	Hispanic	0.97	0.011	0.98	<0.001

* Univariate and multivariable analysis of the demographic and socioeconomic factors predicting the rate of blindness from any cause.

We used variables with p<0.2 in the univariate analyses in an initial multivariable analysis, then we used single backwards elimination to produce the final model shown in the last two columns.

Table 3 shows that blindness from any cause, and blindness from macular degeneration, diabetic retinopathy, congenital anomalies, retinitis pigmentosa, optic nerve atrophy, glaucoma, retinopathy of prematurity, trauma, myopia, corneal/scleral conditions, nystagmus, and other retinal conditions were associated (p < .05 for all) with higher density of ophthalmologists in a univariate model. When adjusting for the predicted risk of blindness based on the socio-economic variables included in the multivariate model (Table 2), only blindness from any cause and blindness from macular degeneration were associated with a greater ophthalmologist density (OR 6.5, p = .003; and OR 15.4, p = .004; respectively).

**Table 3:** People with blindness and density of ophthalmologists.

Condition	Total individuals with Blindness	Not adjusted		Adjusted for expected prevalence of blindness	
		Odds Ratio	p-value	Odds Ratio	p-value
Any Blindness	8350	140.2	<0.001	6.5	0.003
Macular Degeneration	3251	27.0	0.028	15.4	0.004
Diabetic Retinopathy	696	118.4	0.002	1.2	0.827
Congenital Anomalies	662	22.7	0.001	1.6	0.605
Retinitis Pigmentosa	531	24.6	0.013	0.9	0.930
Optic Nerve Atrophy	586	682.2	<0.001	1.2	0.839
Glaucoma	523	1345.9	<0.001	2.7	0.382
Retinopathy of Prematurity	176	829.7	0.003	1.0	0.987
Trauma	167	268.1	0.005	2.3	0.646
Cataract	170	19.0	0.086	1.7	0.727
Myopia	69	1811.2	<0.001	0.4	0.860
Cornea / Sclera	76	33.1	0.044	12.4	0.533
Stargardt’s Disease	14	5628.0	0.111	0.04	0.781
Albinism	10	68.3	0.310	0.08	0.665
Nystagmus	10	90383.4	0.003	37458.0	0.277
Other Retinal Disease	403	64.6	0.001	1.9	0.545
Multiple Syndromes	46	16.5	0.170	5.2	0.415
Other	833	29.6	0.009	1.2	0.877
Unknown	127	28.6	0.143	1.1	0.962

The number of persons with blindness due to various causes, and odds ratios for the change in prevalence of blindness (per 1000 persons in the county) associated with one more **ophthalmologist** per 1000 persons. The first model shows univariate odds ratios; the second model shows the odds ratio after adjusting for expected prevalence of blindness based on the multivariable model predictors.

Table 4 shows that the density of optometrists was not associated with blindness from any cause in a univariate model (p = .511), nor any individual cause except for Stargardt’s disease (p = .046); although the number of observations for this condition was too small (n = 14) to draw definitive conclusions. Macular degeneration was associated with density of optometrists with borderline statistical significance (OR = 5.8, p = .052) in the same multivariable model used to analyze density of ophthalmologists. Similarly, multiple syndromes showed a statistical association (p = .046) in the multivariable analysis, but the sample size was small (n = 46).

**Table 4:** People with blindness and density of optometrists.

Condition	Total individuals with Blindness	Not adjusted		Adjusted for expected prevalence of blindness	
		Odds Ratio	p-value	Odds Ratio	p-value
Any Blindness	8350	0.4	0.511	1.1	0.889
Macular Degeneration	3251	0.2	0.142	5.8	0.052
Diabetic Retinopathy	696	0.3	0.449	0.9	0.879
Congenital Anomalies	662	0.5	0.392	0.5	0.332
Retinitis Pigmentosa	531	0.5	0.525	0.9	0.931
Optic Nerve Atrophy	586	1.6	0.745	2.2	0.324
Glaucoma	523	1.8	0.713	1.0	0.965
Retinopathy of Prematurity	176	2.3	0.685	0.8	0.897
Trauma	167	0.7	0.851	0.4	0.509
Cataract	170	0.2	0.282	0.4	0.475
Myopia	69	151.2	0.016	8.4	0.334
Cornea / Sclera	76	10.1	0.156	5.4	0.284
Stargardt’s Disease	14	600120.5	0.046	3523.6	0.365
Albinism	10	27.1	0.418	15.8	0.531
Nystagmus	10	28.5	0.527	2.2	0.831
Other Retinal Disease	403	1.5	0.778	3.9	0.136
Multiple Syndromes	46	11.7	0.220	62.4	0.046
Other	833	0.4	0.413	0.3	0.126
Unknown	127	1.0	0.991	2.8	0.549

The number of persons with blindness due to various causes, and odds ratios for the change in prevalence of blindness (per 1000 persons in the county) associated with one more **optometrist** per 1000 persons. The first model shows univariate odds ratios; the second model shows the odds ratio after adjusting for expected prevalence of blindness based on the multivariable model predictors.

Fig 1d shows a predictive model for blindness registration within each county described in Table 2 including density of ophthalmologists which was demonstrated to be an independent, significant predictor. This figure identifies several counties with substantially higher odds of blindness than others. For example, Douglas, Josephine, Harney, Lake, and Wheeler have 3-fold higher odds of blindness than counties with the lowest predicted blindness. Examination of the multivariable model suggests that a lower median household income ($39,504) compared to the state median ($46,969), and a lower proportion of Hispanic individuals (5.04%) compared to the state mean (10.85%) were associated with higher registration. In Josephine county, the density of ophthalmologists is double the state average (9.59 vs 4.54/100,000 persons), which may also be a contributing factor for the county higher number of registered individuals. In contrast, Clackamas, Hood River, Morrow, Washington, and Yamhill counties have the lowest proportions of blindness and this may be related to a higher proportion of Hispanic individuals (20.09%) and higher median household income ($58,392 on average). Overall, the results from these analyses suggest that geospatial analytics identified counties with higher and lower than expected proportions of blindness even when adjusted for sociodemographic factors. Clinicians and researchers may use similar methods to identify underserved areas within states to design public health care interventions.

## Discussion

We used a blindness registry to determine the proportion of blindness overall, and the differences in blindness within rural and urban counties. We found a large variation in the proportion of blindness among counties in Oregon, and this proportion was positively associated with the density of ophthalmologists. Blindness was not associated with the density of optometrists. Counties were also more likely to have a higher proportion of blindness when the median household income was lower, the population was older, or the proportion of black individuals was higher. Since blindness registries are available in many states within the US, researchers and public health officials may use the methods and results of this study to better understand the distribution of individuals with blindness and associated factors within states to design public health interventions.

The database included 8350 individuals who were blind in their better seeing eye, which represents 0.21% of the state population. Varma and colleagues [2] estimated blindness in Oregon to be 0.61% of the population over 40 years old, after adjusting for age, sex, and race/ethnicity; which is higher than our estimate. Our estimate may be different because we used the entire population of a county as the denominator because someone of any age could be considered legally blind [2, 26]. If we used those 40 years and older, our estimate would be 0.36%, which is similar to the proportion of the Varma study. The most common causes of irreversible blindness in Oregon were macular degeneration, diabetic retinopathy, congenital anomalies, retinitis pigmentosa, optic nerve atrophy, and glaucoma. This is comparable with the nationwide estimates [27].

Blindness registries are a direct measure of blindness that include certification by an eye care provider, and may be a more accurate than self-report surveys such as the Behavior Risk Factor Surveillance System and the American Community Survey. For example, the American Community Survey’s estimate of blindness is based on a single question, “Is this person blind or does he/she have serious difficulty seeing even when wearing glasses?” [28, 29] While survey questions are low-cost and easy to ascertain, a previous manuscript demonstrated that this question and other survey questions may result in misclassification bias and variability [30, 31]. The Centers for Disease Control and Prevention’s Vision and Eye Health Surveillance System (VEHSS) [27] could consider the methods of the current study to estimate blindness in rural and urban counties as a direct measure.

Our study found associations between blindness and older age (p = .027), black race (p < .001), lower median household income (p < .001), and higher levels of poverty (p < .001). Numerous studies have also demonstrated an association of blindness with these factors [2, 32–39]. A higher proportions of Hispanic individuals was associated with less blindness (p = .01) perhaps their population was younger than other ethnic groups [40]. The association with poverty/lower household income may be attributable to higher unemployment when visually impaired [41–43], poor diet, lack of healthcare insurance, and worse adherence with medical treatments [44–53].

The disparities in eye care availability in Oregon were similar to what Gibson and colleagues [10] estimated for the United States. The average density of ophthalmologists in Oregon was lower than the density of optometrists (4.54 versus 14.88 per 100,000 persons, respectively). These magnitudes are similar to a national estimate that calculated 5.68 ophthalmologists per 100,000 persons across the United States, and 16.16 optometrists in 2015 [54]. Gibson found that the Northeast had the highest number of ophthalmologists per capita (7.6 per 100,000 residents) with the Midwest having the highest number of optometrists per capita (16.1 per 100,000 residents) [10].

In contrast to our hypothesis in which lower access to ophthalmologists would be associated with a higher proportion of blindness, we found the opposite to be true. This association of higher blindness with higher density of ophthalmologists may be related to patients with severe eye disease relocating to areas with a higher density of ophthalmologists. Similarly, this may be an explanation why rural areas were not associated with higher proportions of blindness in that blind individuals migrated to areas with available ophthalmologists. Finally, the association of blindness with ophthalmologist density may be also related to ophthalmologists more commonly detecting and enrolling their patients into a blind registry, i.e. higher detection rates in areas with higher density of ophthalmologists. Wang and Javitt [55] found that in areas with a higher supply of ophthalmologists (greater than 78 per 100,000 persons), individuals with diabetes were 30% more likely to receive an eye examination compared with those in areas with 32 ophthalmologists or fewer. Gibson and colleagues found that individuals who lived in a county within the lowest quartile ophthalmologist availability were significantly more likely to be unaware that they had age-related macular degeneration (ARMD) than individuals who lived in a county in the higher 3 quartiles of ophthalmologist availability [4].

Limitations of this study include that 33.3% of the individuals with blindness did not have geolocation available and needed imputation. However, the data appeared to be missing at random without an association to demographic factors. Most states, like Oregon, use similar registrations to identify and distribute benefits for the blind; however, we do not know the proportion of individuals who are blind, but have not been placed on the blind registry. Similarly, some individuals may apply and be accepted quickly into a state blindness registry, while others may go through an appeal process lasting a year or more [15]. If common, this would lead to underestimation of blindness within a state. Furthermore, the sample size for individual causes of blindness within each county was insufficient to analyze geographical variations in the prevalence of each individual cause of blindness.

## Conclusion

In summary, this study used geospatial statistics to analyze the epidemiology of blindness and the distribution of eye care providers in the state of Oregon. We found a large and statistically significant difference in the proportion of blindness between counties even after adjusting for demographic and socioeconomic factors. A similar approach could be replicated in other states to better understand the distribution of individuals with blindness and the associated factors to help design public health interventions.
